# Inhibition of *Paracoccidioides lutzii Pb*01 Isocitrate Lyase by the Natural Compound Argentilactone and Its Semi-Synthetic Derivatives

**DOI:** 10.1371/journal.pone.0094832

**Published:** 2014-04-21

**Authors:** Renata Silva do Prado, Ricardo Justino Alves, Cecília Maria Alves de Oliveira, Lucília Kato, Roosevelt Alves da Silva, Guilherme Oliveira Quintino, Silvio do Desterro Cunha, Célia Maria de Almeida Soares, Maristela Pereira

**Affiliations:** 1 Laboratório de Biologia Molecular, Instituto de Ciências Biológicas, Universidade Federal de Goiás, Goiânia, Goiás, Brazil; 2 Laboratório de Produtos Naturais, Instituto de Química, Universidade Federal de Goiás, Goiânia, Goiás, Brazil; 3 Núcleo Colaborativo de BioSistemas, Campus Jataí, Universidade Federal de Goiás, Jataí, Goiás, Brazil; 4 Instituto de Química, Departamento de Química Orgânica, Universidade Federal da Bahia, Salvador, Bahia, Brazil; Florida State University, United States of America

## Abstract

The dimorphic fungus *Paracoccidioides* spp. is responsible for paracoccidioidomycosis, the most prevalent systemic mycosis in Latin America, causing serious public health problems. Adequate treatment of mycotic infections is difficult, since fungi are eukaryotic organisms with a structure and metabolism similar to those of eukaryotic hosts. In this way, specific fungus targets have become important to search of new antifungal compound. The role of the glyoxylate cycle and its enzymes in microbial virulence has been reported in many fungal pathogens, including *Paracoccidioides* spp. Here, we show the action of argentilactone and its semi-synthetic derivative reduced argentilactone on recombinant and native isocitrate lyase from *Paracoccidioides lutzii Pb*01 (*Pb*ICL) in the presence of different carbon sources, acetate and glucose. Additionally, argentilactone and its semi-synthetic derivative reduced argentilactone exhibited relevant inhibitory activity against *P. lutzii Pb*01 yeast cells and dose-dependently influenced the transition from the mycelium to yeast phase. The other oxygenated derivatives tested, epoxy argentilactone and diol argentilactone**-**, did not show inhibitory action on the fungus. The results were supported by *in silico* experiments.

## Introduction

The dimorphic fungus *Paracoccidioides* spp. is the causative agent of paracoccidioidomicosis (PCM), the most prevalent invasive fungal disease in South America [1]. PCM is responsible for more than 50% of the deaths due to fungal infections [2]. There are distinct forms of PCM [3], and treatment regimes of long duration are required for the maintenance of patients with the more severe forms; however, relapses remain a significant unresolved problem [4]. Cases of PCM associated with AIDS have also been reported [5], [6]. The majority of the clinically used antifungal drugs have various drawbacks in terms of toxicity, efficacy, and cost, and their frequent use has led to the emergence of resistant fungal strains [7].

New therapeutic approaches for PCM have been performed [8], and natural compounds with antifungal activity against *Paracoccidioides* spp. have been evaluated [9]. Nonetheless, there is still a great demand for novel antifungal agents belonging to a wide range of structural classes and acting selectively on novel targets with fewer side effects. With this focus, our group has investigated the enzymes 1,3-β-D-glucan synthase (*Pb*FKS1) [10], [11], malate synthase (*Pb*MLS) [12], [13], isocitrate lyase (*Pb*ICL) [14], and (S)-adenosyl-L-methionine: Δ24 sterol methyl transferase (*Pb*SMT) [15] from *Paracoccidioides lutzii Pb*01.

Since expression of glyoxylate cycle genes malate synthase and isocitrate lyase are detected during specific stages of the interaction between host and pathogen in a variety of human-pathogenic bacteria and fungi [16], [17], [18], [19], [20], [21], [22], [23], [24], [25], [26], [27], [28], [29], including in *Paracoccidioides* [30], [31], [32], [33], [34], the development of specific inhibitors against ICL is an attractive prospect. Moreover, drugs inhibiting ICL would be predicted to have less toxicity because it is not found in mammals. Although some isocitrate lyase (ICL) inhibitor compounds tested against *Candida albicans* and *Mycobacterium tuberculosis* have been described in the literature [35], [36], no inhibitor for *Pb*ICL has been reported to date. *Pb*ICL transcripts are highly abundant in *P. lutzii Pb*01 yeast cells [37], and up-regulation of this gene occurs during the transition from mycelium to yeast [33] and during the infection process [34] and internalization by macrophages [32]. In addition, the inactivation of *Pb*ICL by phosphorylation is reversible, indicating a new strategy for the rapid adaptation to changing environmental conditions [14]. These findings support the importance of searching for *Pb*ICL inhibitors.

There are abundant natural compounds from the Brazilian Savannah flora endowed with antifungal activity. Thus, in an effort to identify agents active against *Pb*ICL, we focused on argentilactone, the major constituent of the essential oil of *Hyptis ovalifolia*, as this compound is known to suppress the proliferation of such microorganisms as *Cryptococcus neoformans*, *C. albicans*, and the dermatophytes *Microsporum canis, Microsporum gypseum, Trichophyton mentagrophytes*, and *Trichophyton rubrum* [38]. The argentilactone and its semi-synthetic derivatives were selected based on the structural similarities with itaconic acid [39] and the sesterterpene halisulfate [19], [40], both described in the literature as ICL inhibitors.

Herein, we report the inhibitory action of argentilactone and its semi-synthetic derivatives reduced argentilactone, epoxy argentilactone, and diol argentilactone on *P. lutzii Pb*01 yeast cells, during the differentiation from mycelium to yeast, and on recombinant and native *Pb*ICL enzymes in the presence of different carbon sources. *In silico* analyses were performed to corroborate the *in vivo* studies.

## Results and Discussion

### Chemistry

**Figure 1:**
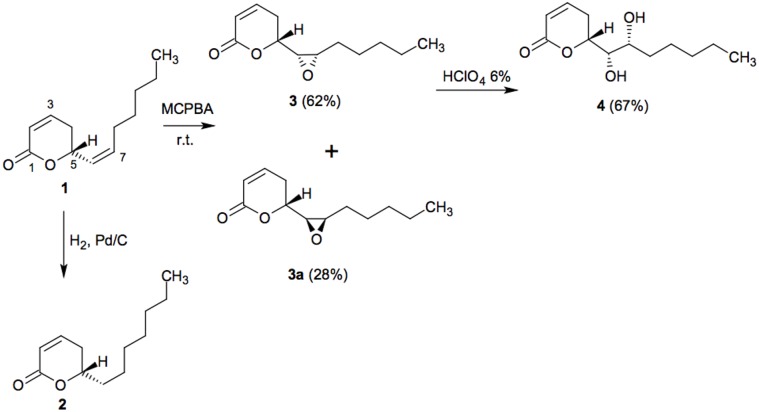
Structures and synthesis of compounds reduced argentilactone (2), epoxy argentilactone (3 and 3a) and diol argentilactone (4) from natural compound argentilactone (1).

Compounds reduced argentilactone (2), epoxy argentilactone (3) and diol argentilactone (4) were synthesized efficiently using simple and well-established reactions (Fig. 1). The hydrogenation reaction of natural compound argentilactone using Pd/C led to reduced argentilactone. The oxidation reaction of argentilactone with *m*-chloroperoxybenzoic acid was visualized as a means of obtaining the epoxides **3** and **3a** through electrophilic attack exclusively at the isolated double bond. The major epoxide **3** was stirred in HClO_4_ solution to furnish the diol **4**.

### Effect of Argentilactone and its Derivatives Reduced Argentilactone, Epoxy Argentilactone, and Diol Argentilactone on *P. Lutzii Pb*01 Yeast Cells Growth

The activity of argentilactone from *H. ovalifolia* and its derivatives against *P. lutzii Pb*01 yeast cells was verified in this study. Considering that pathogenic microorganisms utilize different carbon sources during pathogenesis [20] and that *Pb*ICL is regulated by the carbon source, we evaluated the *P. lutzii Pb*01 yeast cells growth using glucose or acetate as the carbon source in the presence of argentilactone, reduced argentilactone, epoxy argentilactone, and diol argentilactone.

**Figure 2:**
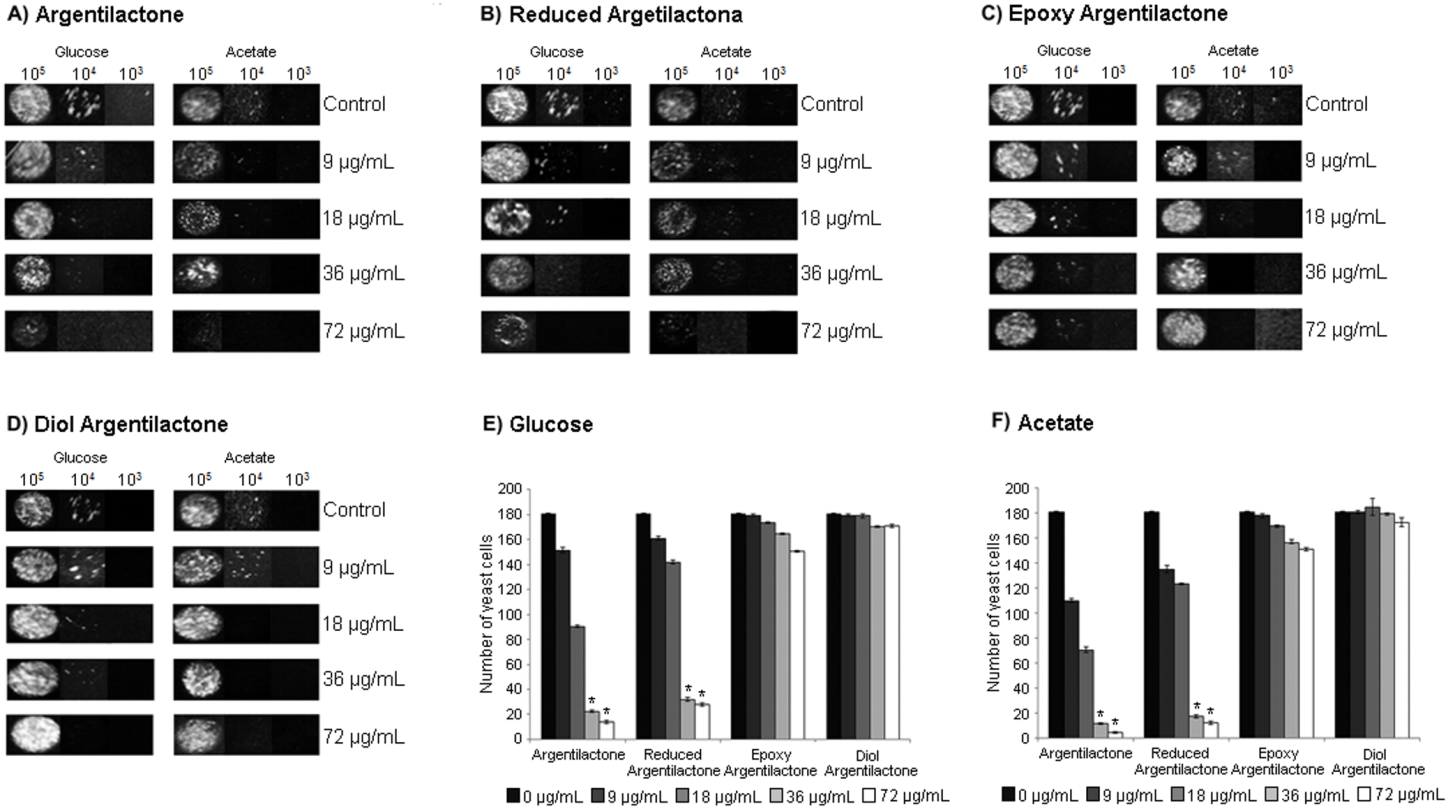
Effect of argentilactone and its derivatives reduced argentilactone, epoxy argentilactone, and diol argentilactone on *P. lutzii Pb*01 yeast cells growth. Samples containing 10^5^, 10^4^, and 10^3^ *Paracoccidioides Pb*01 yeast cells were spotted onto MMcM agar medium containing glucose or acetate and supplemented with argentilactone, reduced argentilactone, epoxy argentilactone, and diol argentilactone at different concentrations for seven days. **A**) argentilactone; **B**) reduced argentilactone; **C**) epoxy argentilactone; **D**) diol argentilactone. The growth of 10^5^ *P. lutzii Pb*01 yeast cells was observed by spectrophotometer (520 nm) in medium containing glucose (**E**) or acetate (**F**). *p<0,05.

**Figure 3:**
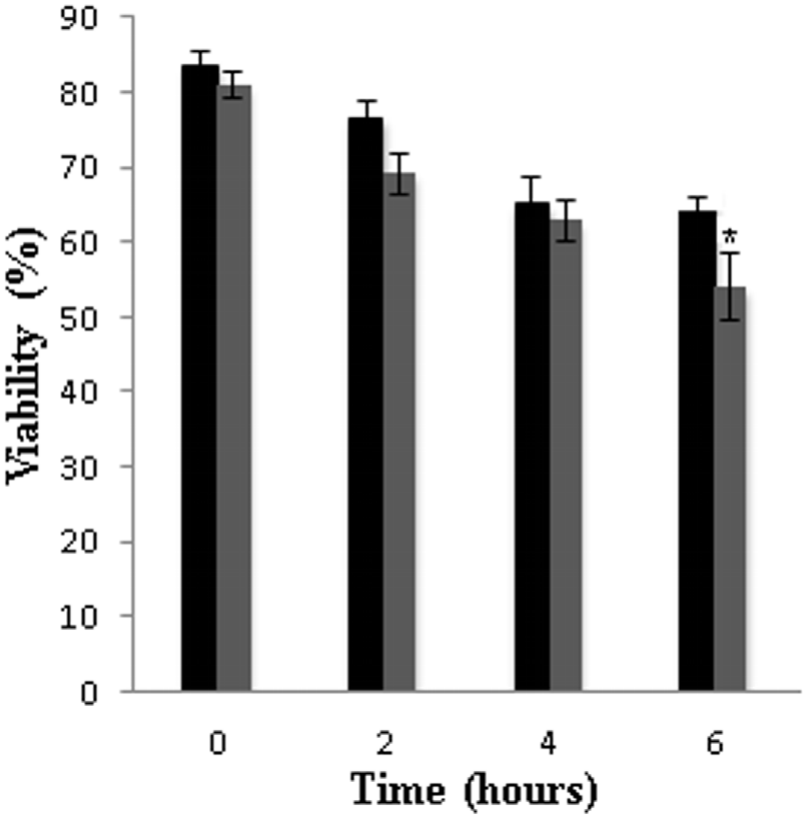
Effect of argentilactone on *P. lutzii Pb*01 yeast cells growth. Yeast cells were cultured at 36°C in the presence of argentilactone for 6 hours. Aliquots were taken and the cells were counted in a Neubauer chamber.*p<0,05.

The results showed a dose-dependent inhibition by argentilactone and reduced argentilactone on *P. lutzii Pb*01 yeast cells growth when the fungus was cultured in the presence of glucose or acetate as the carbon source (Fig. 2A and B, respectively). A very small effect on fungal growth was observed for epoxi and diol argentilactone (Fig. 2C and D, respectively) that was independent of the carbon source (Fig. 2E and F). In contrast, the growth inhibition by argentilactone and reduced argentilactone was influenced by the carbon source (Fig. 2E and F). The minimal inhibitory concentration was lower in the presence of acetate (9 µg/mL **1** and **2**) (Fig. 2F) than glucose (18 µg/mL **1** and **2**) (Fig. 2E). Argentilactone was more effective against *P. lutzii Pb*01 yeast cells than dermatophytes, as the minimal inhibitory concentration values were lower than those reported for dermatophytes (31 µg/mL) [38]. Viability assay of *P. lutzii Pb*01 exposed to argentilactone **(**18 µg/mL)**,** showed that after 6 h of incubation there was a decrease of approximately 15% viability (Fig. 3). Argentilactone **(**18 µg/mL) was not toxic to MRC5 cells (data not show).

### Effect of Argentilactone and its Derivatives Reduced Argentilactone, Epoxy Argentilactone, and Diol Argentilactone on Phases of *P. Lutzii Pb*01

In the soil, *P. lutzii Pb*01 grows as a saprophytic mycelium, resulting in the formation of propagules that initiate the infection in humans when inhaled into the respiratory tract; the mycelial propagules subsequently develop into yeast cells in the lung. The transition is important for infection and disease [41] and can be replicated *in vitro* [42]. Bastos *et al*. [33] showed that the transcript levels of the *Pb*ICL gene in this fungus increase during the mycelium to yeast transition. Considering that the carbon source used regulates *Pb*ICL expression, we evaluated whether argentilactone and its derivatives reduced argentilactone, epoxy argentilactone, and diol argentilactone interfered with the dimorphic transition of *P. lutzii Pb*01 from mycelium to yeast when cultured in the presence of glucose or acetate.

**Figure 4:**
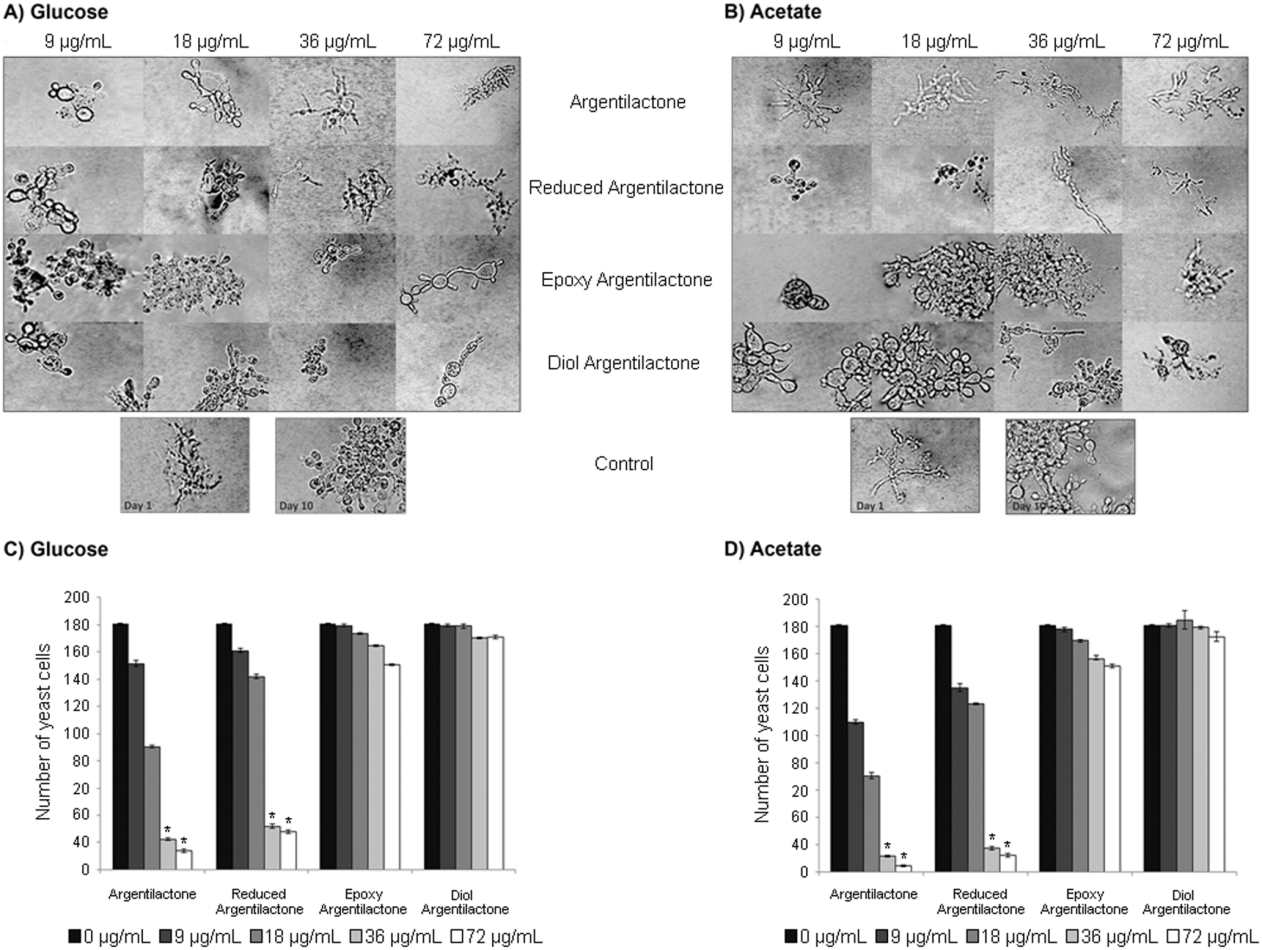
Effect of argentilactone, reduced argentilactone, epoxy argentilactone, and diol argentilactone on *P. lutzii Pb*01 differentiation from mycelium to yeast. The mycelium was incubated for 10 days at 36°C on MMcM agar containing glucose or acetate and supplemented with different concentrations of argentilactone, reduced argentilactone, epoxy argentilactone, and diol argentilactone. Cell morphology was observed by optical microscopy in medium containing glucose (**A**) or acetate (**B**). The *P. lutzii Pb*01 yeast cells were counted using a Neubauer chamber in medium containing glucose (**C**) or acetate (**D**). *p<0,05.

The results showed a dose-dependent inhibition by argentilactone of the dimorphism of *P. lutzii Pb*01 when the fungus was cultured in the presence of glucose or acetate (Fig. 4). However, at ten days after changing the cultivation temperature from 23°C to 36°C, the dimorphism inhibition was greater in the presence of acetate than glucose. Similar results were found for reduced argentilactone, though the effect was reduced, whereas derivatives epoxi and diol argentilactone did not interfere with the dimorphism process. The higher inhibition by argentilactone and reduced argentilactone on *P. lutzii Pb*01 yeast cells growth and dimorphism in the presence of acetate versus glucose suggests a high specificity of these compounds for *Pb*ICL.

Most microorganisms that are capable of utilizing acetate or other fatty acids as the sole carbon source employ the glyoxylate cycle for the anaplerosis of oxaloacetate, which is dispensable during growth on glucose. Therefore, the glyoxylate cycle and the tricarboxylic acid (TCA) cycle must operate simultaneously because some reactions from the glyoxylate cycle are shared with the TCA cycle [43], [44]. The key enzymes of the glyoxylate cycle consist of ICL and malate synthase, which are assumed to function solely in the glyoxylate cycle [45]. Although *Pb*ICL activity was found to be higher on two-carbon sources, it is also detectable on glucose [14]. The interference of the argentilactone on the *Pb*ICL activity was evaluated on mycelium, during differentiation from mycelium to yeast, and on yeast phase (Table 1). The argentilactone inhibited *Pb*ICL on all conditions. The inhibition was higher on acetate (∼45%) than glucose (∼30%). The *Pb*ICL activity was higher on yeast phase, differentiation, and mycelium, respectively. Those results are in according with the higher level of *Pb*ICL transcript and protein during differentiation when compared to mycelium [33]; [31].

**Table 1:** Inhibitory effect of the argentilactone on the *Pb*ICL activity from phases of *Paracoccidioides lutzii Pb*01.

Mycelium		Mycelium to Yeast		Yeast	
*Glucose*	*Acetate*	*Glucose*	*Acetate*	*Glucose*	*Acetate*
**C** 0.055±0.003	0.075±0.001	0.075±0.001	0.120±0.001	0.128±0.002	0.250±0.001
**T** 0.045±0.001	0.055±0.005	0.055±0.001	0.055±0.003*	0.085±0.001*	0.120±0.003*

The values correspond to specific activity in U = 1 µmol of glyoxylate-phenylhydrazone per minute;

C: Control without argentilactone;

T: Treated with 18 µg/mL of argentilactone;

*p<0.05.

### Inhibitory Effect of Argentilactone and its Derivatives Reduced Argentilactone, Epoxy Argentilactone, and Diol Argentilactone on *Pb*ICL Activity

A crude extract of *Paracoccidioides Pb*01 yeast cells was obtained to investigate the action of argentilactone and its derivatives reduced argentilactone, epoxy argentilactone, and diol argentilactone on native *Pb*ICL activity. Argentilactone and reduced argentilactone inhibited *Pb*ICL activity, as shown in Table 2. A higher inhibition was observed in the presence of acetate (50% inhibition; IC_50_ = 50 µM) than glucose (20% inhibition; IC_50_ = 80 µM), possibly due to the high level of *Pb*ICL inactivity on glucose, which would bind to argentilactone and reduced argentilactone. No inhibition was observed for epoxi or diol argentilactone. The post-translational regulation of *Pb*ICL depending of carbon source could be one of the explanations to the high level of *Pb*ICL inactivity on glucose. According to Cruz *et al.* [14], although the total *Pb*ICL protein level in glucose-grown cells is slightly lower than in acetate-grown cells, the *Pb*ICL specific activity in yeast cells grown on glucose is much lower than would be expected based on the protein abundance due post-translational regulation of enzymatic activity. The action of argentilactone and derivatives reduced argentilactone, epoxy argentilactone, and diol argentilactone was also investigated using recombinant isocitrate lyase from *Paracoccidioides Pb*01 (*Pb*ICLr). *Pb*ICLr inhibition was higher in the presence of argentilactone (IC_50_ = 28.8 µM) than reduced argentilactone (IC_50_ = 30.2 µM). In contrast, epoxy argentilactone and diol argentilactone had no effect against *Pb*ICLr.

**Table 2:** Inhibitory effect of the compounds on the *Pb*ICL activity.

Compound		Specific activity (U./mg)		IC_50_ (µM)		
	*Pb*ICLr	*Pb*ICLc#		*Pb*ICLr	*PbICLc#*	
		*Acetate*	*Glucose*		*Acetate*	*Glucose*
Control	2.55±0.02	0.080±0.004	0.010±0.002			
3-Nitropropionate	0.81±0.01*	0.036±0.002*	0.008±0.001	25.7±0.01	45.4±0.01	76.0±0.01
Argentilactone	0.86±0.01*	0.040±0.001*	0.008±0.asdf003	28.8±0.01	50.0±0.03	80.0±0.02
Reduced argentilactone	0.93±0.01*	0.067±0.004	0.009±0.003	30.2±0.04	60.0±0.02	85.0±0.01
Epoxi argentilactone	N.I.**	N.I.**	N.I.**	N.I.**	N.I.**	N.I.**
Diol argentilactone	N.I.**	N.I.**	N.I.**	N.I.**	N.I.**	N.I.**

# *Pb*ICL activity in the crude protein extract of *Paracoccidioides lutzii Pb*01 yeast cells;

. U = 1 µmol of glyoxylate-phenylhydrazone per minute;

* *p*<0.05;

**N.I. = No inhibition found.

### Homology Modeling

Because there is no experimentally resolved 3D structure for *Pb*ICL thus far, the amino acid sequence was compared to sequences in Brookhaven Protein Data Bank (PDB) using the BlastP program [46]. *Aspergillus nidulans* ICL (*An*ICL) (PDB code: 1dqu) was found to be 85% identical to *Pb*ICL and thus was selected as the best template among the structures deposited in PDB.

**Figure 5:**
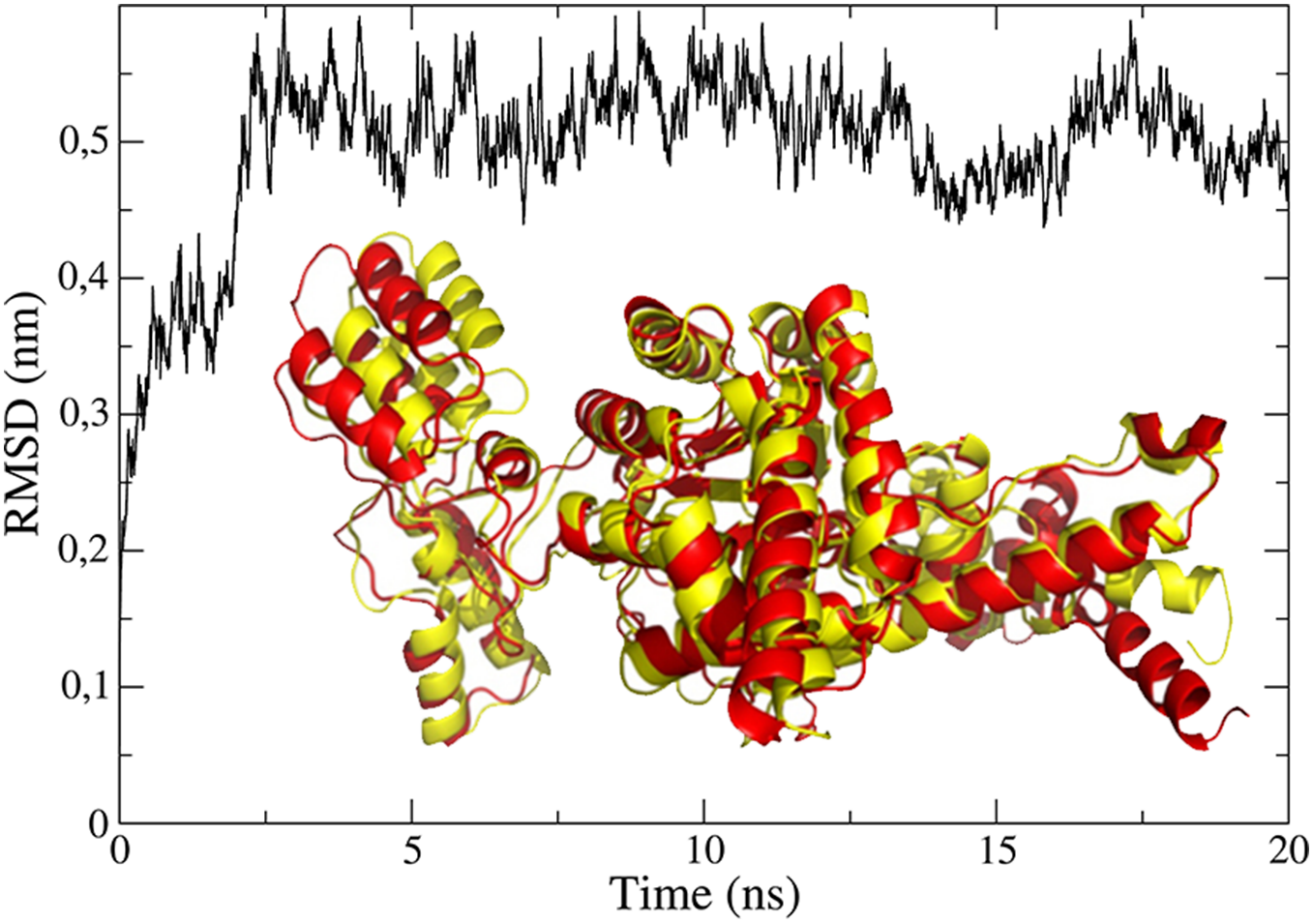
Dynamic profile for RMSD obtained in molecular dynamic simulations of *Pb*ICL after 20 ns. Superimposition of the homology model (red) and molecular dynamic structure (yellow) of *Pb*ICL is shown. The molecular dynamic-model structure was obtained using the program g_cluster, which determined the conformation that best represents the last 10 ns of the trajectory.

The homology model of *Pb*ICL showed very little conformational change when compared to the template *An*ICL. *Pb*ICL had 0.49 Å of root-mean-square deviation (RMSD) when superimposed with respect to the non-hydrogen atoms, and significant conformational changes were only observed for the N-terminal and C-terminal regions (Fig. 5). The patterns of secondary structures are also very similar with respect to the proportions: an alpha-helix-like pattern corresponds to more than 50% of the entire structure, with a less than 10% beta-sheet-like pattern (data not shown).

The homology model of *Pb*ICL was assessed stereochemically using a Ramachandran plot. The Φ and Ψ distributions of the Ramachandran plots showed 97.3% and 1.7% of the residues in favored and allowed regions, respectively; only 1% of the residues are outside the allowed region. The structure quality factor was 88.2, as estimated by ERRAT [47].

### Molecular Dynamics

Molecular dynamic simulations of the *Pb*ICL structure were performed to improve the relaxation and orientation of their side chains. This procedure is usually crucial when reproducing the structural stability of the receptor in its native environment [48]. The *Pb*ICL structure remained stable at approximately RMSD = 0.5 nm after approximately 5 ns of simulation. Although there were fluctuations in the RMSD, mainly from 12 ns, only one particular conformation was required to represent the set of conformations, as detected using the g_cluster program. That conformation was then selected for the subsequent studies (Fig. 5).

The selected structure of *Pb*ICL after the molecular dynamic simulations showed an RMSD increase from the homology model to approximately 4.32 Å after 20 ns. However, the RMSD increased by only 1.61 Å when only residues involved in the binding pocket (defined in the next section) were considered. The conformation of the binding pocket was preserved after the molecular dynamic simulations. More pronounced deviations were observed in sections involving C-terminal residues from ASN500 and, in particular, between PRO269 and ALA300. However, those segments are far outside the region of the binding pocket and could not influence the interaction between a ligand and the residues involved in this region of the protein. Differences in the secondary structure content were rarely observed between the homology and molecular dynamic models, with only higher helix content from residue ALA452 being observed in the homology model.

### Molecular Docking

Simulations involving the search for conformations with the substrates on the surface of *Pb*ICL were limited to only a region surrounding the binding pocket of the protein, which was defined as involving the same residues of the template provided by [49]. Amino acids within the *Pb*ICL binding site (ASP24-GLN55 and ILE227-THR236) were 100% identical to the template *An*ICL.

Initially, molecular docking between *Pb*ICL and isocitrate was conducted to evaluate its energy profile in relation to the *Pb*ICL binding pocket. As expected for an original ligand, a very specific energy was found, Fscore =  −6.8 Kcal/mol for 100% of the simulations (Table 3), showing that, in fact, isocitrate is very well defined for the *Pb*ICL binding pocket and concomitantly indicating that the structure predicted by molecular dynamic is consistent with the expected structure.

**Table 3:** Scores and key residues of the compounds obtained in binding pocket of *Pb*ICL.

Ligands	Active torsions	Fscore^1^ (Kcal/mol)	Key residues
Argentilactone	6	−6.9	ASN 53; GLY 235; LYS 47
Epoxi argentilactone	5	−6.asdf8	ASN 53; GLY 235; LYS 47
Diol argentilactone	8	−7.4	ASN 53; ASP 237; GLY 235; LYS 47; LYS 57; TRP 21
Isocitrate	2	−6.8	ARG 43; LYS 47

^1^Modified Vina Score (Eq. 01). The values in the table represent the states with the highest frequency of total of 1000 independent simulations.

**Figure 6:**
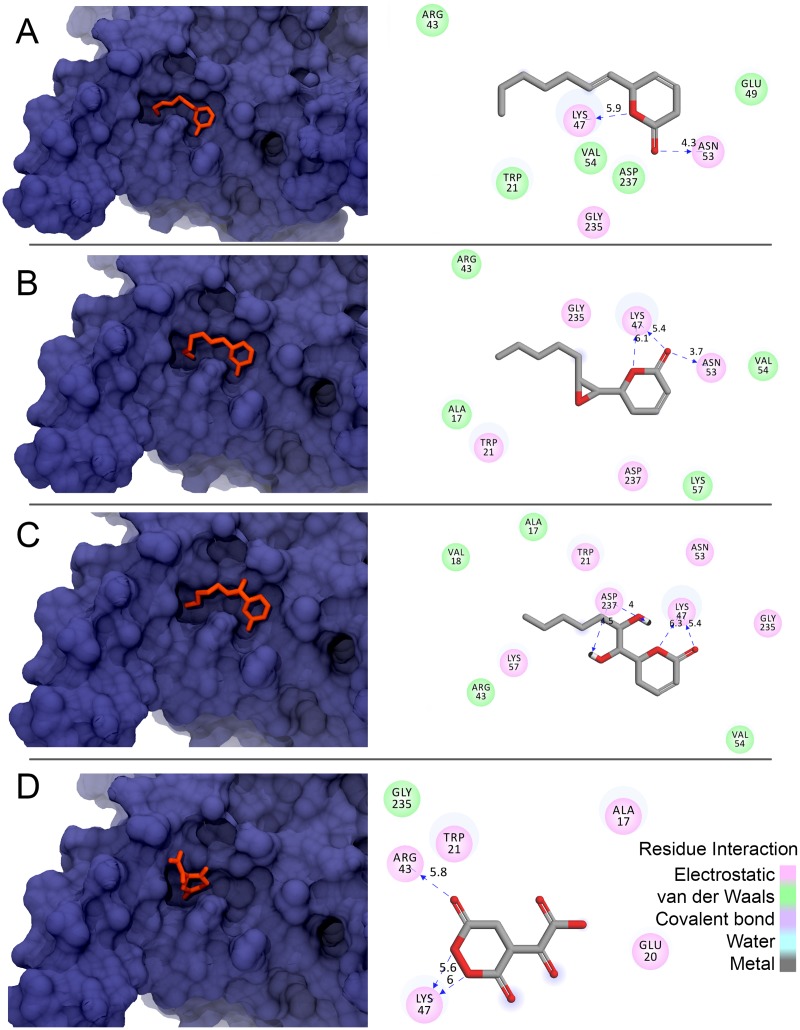
Molecular surface representation of PbICL and the three-dimensional structures of A) 1, B) 3, C) 4, and D) isocitrate (native ligand) in the PbICL binding pocket and key interactions of PbICL with the best conformation (mode 1) of each compound (orange). Ligplots of the compounds in binding pocket of PbICL. Pink circles indicate residues involved in hydrogen-bond polar or charged interactions; green circles indicate residues involved in van der Waals interactions. Dashed lines represent stronger interactions. The PbICL residues that interact with the compounds are shown, and the dashed lines represent stronger interactions. Oxygen is colored red. The structures of the compounds shown refer to the lowest Fscore obtained in mode 1 from the docking simulations with AutoDock Vina. The grid was defined considering only the region of the PbICL site using sizex = sizey = sizez = 20 Å, centerx = 21.37 Å, centerx = 1.14 Å, and centerz = 10.83 Å.

Because argentilactone and reduced argentilactone are identical in the model involving only hydrogen atoms, polar docking simulations were only performed using argentilactone. With the exception of diol argentilactone, with an Fscore reaching −7.4 Kcal/mol, the other compounds (argentilactone, epoxy argentilactone, and diol argentilactone) also had Fscores of approximately −6.8 Kcal/mol, but the energies were less specific, ranging from conformations with an Fscore around or slightly above this value. Figure 6 shows how each compound is accommodated in the *Pb*ICL binding pocket and the *Pb*ICL residues interacting more strongly with each of the compounds. Note that the oxygen of the ester group present in argentilactone, reduced argentilactone, and diol argentilactone is accommodated in the *Pb*ICL binding pocket in a position favoring the formation of a hydrogen bond with the amine group of ASN53. In the case of reduced argentilactone and isocitrate, the heterocyclic moiety is in another part of the cavity, located laterally to the anterior region, whereby the oxygen atoms of the heterocyclic moiety form hydrogen bonds with the amine group of ARG43. In addition, except to isocitrate, the aliphatic chain moieties of other compounds can be accommodated in the *Pb*ICL binding pocket. In particular, the carbonyl group of diol argentilactone favorably contributes to the formation of hydrogen bonds with the ammonium group of LYS57.

Table 3 lists the key residues involved in the interaction with each of the compounds. For isocitrate, the key residues are LYS47 and ARG43, which are preserved between the template and *Pb*ICL. The ammonium group of LYS47 is involved in the interaction with all the compounds. ARG43 appears only to stabilize isocitrate; in the other compounds, this interaction is replaced by ASN53 and GLY235. The ammonium portion of TRP21 is also involved in the interactions with reduced argentilactone and diol argentilactone.

Mutations involving the ASN53, GLY235 and LYS47 residues were performed to evaluate the importance of the stereochemistry of each one of these residues to provide stability to argentilactone. The multi-point mutations in the key amino acid residues were performed successfully using Swiss-PDB viewer. The point mutations were chosen strategically to dramatically change the size and nature of their side chains to affect the binding pocket. The effect of these mutations on stability of argentilactone compared to the original binding pocket (native *Pb*ICL) can be seen in Supplementary Figure S1. Note that both mutations involving ASN53 or GLY235 appreciably affect the values of Fscore. However, for the mutation involving LYS47 residue, the behavior of Fscore argentilactone is quite similar to non-mutated state. This reinforces the hypothesis that argentilactone is much more stable due to its hydrophobic content than due to a specific setting its polar interactions with *Pb*ICL.

In fact, isocitrate has only two active rotatable bonds (Table 3), which contributes to its better specificity in the *Pb*ICL binding pocket when compared to the other compounds studied. In addition, compounds with a smaller number of atoms have been considered to be more efficient ligands [50]. Argentilactone and its derivatives achieve more than five active rotating bonds, and argentilactone can easily be accommodated as a ligand on the surface due to its highest hydrophobic content, with a lower Fscore, but otherwise providing a less defined Fscore distribution (data not shown).

### Intermolecular Interactions and Solvation Free Energies

The intermolecular energy between *Pb*ICL and the compounds was also estimated using molecular dynamic simulation-constrained conformations of the protein and the ligand so that the conformation predicted by AutoDock Vina [51] could be preserved, thus allowing an evaluation of the major solvent contributions around these states. Table 4 shows the non-bonded potential energy (Lennard-Jones and Coulomb contributions) involved in the receptor-ligand and ligand-solvent interactions. Argentilactone has the lowest Lennard-Jones energy (receptor-ligand), in this case resulting in the lowest value of accessible surface area (ASA). Isocitrate has a potential energy of almost −30 Kcal/mol, which is less than argentilactone, though the ASA is more than five times available to the solvent, as evidenced by the energy difference observed for the short-range Coulomb contributions involving the ligand-solvent interaction.

**Table 4:** Intermolecular energies of the compounds in binding pocket of *Pb*ICL.

Ligands	Intermolecular energies receptor (Kcal/mol)		Intermolecular energies solvent (Kcal/mol)		Potential energy^3^ (Kcal/mol)	ASA^a^ (Å^2^)	ΔG_solv_ (Kcal/mol)
	LJ_SR^1^	Coul. SR^2^	LJ_SR^1^	Coul. SR^2^			
Argentilactone	−84.5±0.6	−20.0±0.6	−27.9±0.1	−1.9±0.7	−134.3	90 (388§)	−16.2±1.1
Epoxi argentilactone	−79.0±1.5	−6.3±0.5	−30.3±0.4	−1.0±0.4	−116.6	106 (405)	−29.8±1.6
Diol argentilactone	−77.5±1.0	−13.0±4.4	−46.6±0.5	−11.3±3.9	−152.0	105(423)	−18.6±1.5
Isocitrate	−68.9±1.7	−24.9±3.0	−26.4±0.5	−44.3±2.9	−164.5	511 (1475)	−60.7±1.2

^1^Lennard-Jones Energies inside the shortest cutof (Short-Range);

^2^Coulomb potential within rcoulomb cutof (Short-Range);

^3^non-bonded terms only were considered;

^a^Accessible Surface Area (ASA) to the solvent in binding pocket of *Pb*ICL.

§ Accessible Surface Area to the solvent of the compound without the presence of *Pb*ICL, but maintaining its conformation conferred in the binding pocket.

The solvation free energies ΔG_solv_ of each compound were estimated using thermodynamics perturbation methods (see the Experimental section). The ΔG_solv_ values for the compounds and the magnitude of the error in evaluating ΔG_solv_ are reported in Table 4. The deviations ranged from −1.1 to −1.8 Kcal/mol, representing percentage errors ranging 2.0–7.0%.

Almost four times more free energy is necessary to desolvate isocitrate (−60.7 Kcal/mol) in comparison to argentilactone (−16.2 Kcal/mol) (Table 4), indicating that argentilactone situated in the *Pb*ICL binding pocket more favorably contributes to the increase in the entropy of the medium. Derivative diol argentilactone also has a ΔG_solv_ very close to that observed for argentilactone. However, considering the amount of ASA in the *Pb*ICL binding pocket and the ΔG_solv_ proportional to this area, only 1 Kcal/mol more energy would be required to desolvate diol argentilactone compared to argentilactone.

The *Pb*ICL binding pocket favors the formation of several hydrogen bonds between isocitrate-*Pb*ICL and isocitrate-solvent. Isocitrate is the most polar among the compounds, therefore a great difference in ΔG_solv_ can be observed in relation to the other compounds (Table 4). Consequently, the isocitrate ASA is four times greater than other compounds. Although argentilactone does not have the lowest energy among the intermolecular compounds (see the potential energies in Table 4), the binding free energy depends on the path that the compound adopts when binding to the *Pb*ICL pocket. In this case, diol argentilactone and epoxy argentilactone may require much more specificity than argentilactone and thus may contribute adversely to the binding free energy. Argentilactone requires little energy for desolvation when compared to the other compounds (see ΔG_solv_ in Table 4). Indeed, the hydrophobic groups ensure an appreciable increase in the entropy of the medium when this ligand is well accommodated in the *Pb*ICL binding pocket, i.e., when there is good complementarity. This fact can be guaranteed, as argentilactone has the lowest ASA (90 Å^2^), and the nonpolar groups are very well protected in the *Pb*ICL binding pocket (Fig. 6A).

Our hypothesis is that torsion and the nonpolar content of argentilactone can provide sufficient flexibility to increase its specificity and facilitate accessibility to the *Pb*ICL binding pocket, ensuring a lower binding free energy (not estimated in this study). This hypothesis could be evaluated to estimate the ligand dissociation path to calculate the smooth reaction path that links the bound and unbound states. In our next study, these estimates may be determined using umbrella sampling to calculate the potential of mean force and estimate the binding free energy of each compound.

## Conclusion

Our *in vivo*, *in vitro*, and *in silico* results document the inhibitory activities of compound argentilactone and its derivatives reduced argentilactone, epoxy argentilactone, and diol argentilactone on *P. lutzii Pb*01. Argentilactone inhibited *Pb*ICL activity, *P. lutzii Pb*01 yeast cells growth, and differentiation from mycelium to yeast. The lowest inhibition was found for reduced argentilactone when compared to argentilactone; the lack of inhibition by epoxy argentilactone and diol argentilactone is most likely due to the change in the polarity of the molecules. *In silico* analyses allowed an assessment of the important aspects of the stability and kinetics of the compounds for accommodation in the binding pocket. The characteristics observed for argentilactone indicate its ability to be accommodated in the *Pb*ICL binding pocket, suggesting a higher stability in relation to the other compounds. Therefore, our results indicate that argentilactone is an excellent candidate as an antifungal model.

## Materials and Methods

### Experimental Chemistry

NMR spectra were recorded with a Varian Mercury plus BB spectrometer operating at 300 MHz for ^1^H and at 75.457 MHz for ^13^C. CDCl_3_ was used as the solvent, with Me_4_Si (TMS) used as the internal standard. TLC was performed using precoated Kiesegel 60 F254 plate (Merck and M. Nagel). The spray reagent used for TLC was 5% anisaldehyde in ethanol [52].

### Extraction of Argentilactone (2H-Pyran-2-one, 6-(1-heptenyl)-5,6-dihydro-, [R-(Z)])(1)

The essential oil of *H. ovalifolia* was obtained from fresh leaves by hydrodistillation in a Clevenger-type apparatus for 5 h. The aqueous phase was extracted with diethyl ether, and the organic phase was dried over anhydrous sodium sulfate and concentrated under N_2_ to yield 0.05% of the essential oil based on the fresh leaf weight. The identification of the compounds was based on the comparison of their retention indices and mass spectra with data in the literature and through computerized matching of the acquired mass spectra with the GC/MS data system. Separation of argentilactone was performed by CC using silica gel (Merck) and hexane - ethyl ether (4∶1). Argentilactone (**1**): [α]_D_: −21° (*c* 2.0, CHCl_3_) (literature value of −21° (EtOH)). IR (KBr) ν^max^/cm^−1^: 2928, 1723, 1380. EIMS *m/z* (rel. int.): 194 [M]+· (3), 97 (20), 68 (100); ^1^H NMR (CDCl_3_, 300 MHz): Δ H 0.89 (t, J = 6.6 Hz, 3H, H-12), 1.40–1.30 (m, 6H, H-9, H-10 and H-11), 2.10 (m, 2H, H-8), 2.40 (m, 2H, H-3), 5.22 (ddd, J = 10.2, 8.4 and 5.5 Hz, 1H, H-5), 5.56 (ddt, J = 11.1, 8.4 and 1.5 Hz, 1H, H-6), 5.66 (dtd, J = 11.1, 7.5 and 0.9 Hz, 1H, H-7), 6.05 (ddd, J = 9.8, 2.4 and 1.2 Hz, 1H, H-2), 6.90 (ddd, J = 9.8, 5.4 and 3.0 Hz, 1H, H-3). 13C NMR (CDCl3, 75.4 MHz) Δc 13.7 (C-11), 27.5 (C-8), 28.8 (C-9), 29.6 (C-4), 31.1 (C-10), 73.7 (C-5), 121.6 (C-2), 126.3 (C-6), 135.6 (C-7), 145.0 (C-3), 164.3 (C-1). These data are consistent with the literature [38].

### Synthesis of Compound Reduced Argentilactone (2*H*-Pyran-2-one, 6-heptyl-5,6-dihydro-, (6*R*)) (2) from Argentilactone

The reduction of argentilactone was performed using a stirred solution of argentilactone (0.8 g, 4.1 mmol) in ethanol (10 mL), 8 mg of Pd/C under H_2_ (1 atm) and the mixture was allowed to a stand at room temperature for 3 h. The solvent was evaporated. Chromatography of the crude product afforded the compound reduced argentilactone (0.60 g, 3.0 mmol).

### Synthesis of Compound Epoxy (2*H*-Pyran-2-one, 5,6-dihydro-6-[(2*S*, 3*S*)-3-pentyl-2-oxiranyl] -, (6*R*)) (3) and Epoxy (3a) from Argentilactone

The epoxidation of argentilactone was performed according Alves [52] *m*-cloroperbenzoic acid (2.3 g) was added to a stirred solution of argentilactone (1.5 g, 7.7 mmol) in dry CH_2_Cl_2_ (15 mL), and the mixture was allowed to a stand at room temperature for 2 days. The CH_2_Cl_2_ solution was washed with 10% aqueous NaHCO_3_ (3×25 mL), dried (Na_2_SO_4_), and evaporated. Chromatography of the crude product afforded the mixture of epoxides **(3)** (1.0 g, 4.7 mmol) and epoxy **(3a)** (0.46 g, 2.2 mmol). Epoxide (**3**) (yield 62%). IR (KBr) ν^max^/cm^−1^: 2936, 2847, 1734, 1379, 1245, 1042, 808 cm^−1^. ^1^H NMR: CDCl_3_, 500 MHz Δ_H_ 0.90 (*t*, 3H, H-12), 1.34–1.67 (*m*, 8H, H-8, H-9, H-10, H-11), 2.60 (*m*, 2H, H-4), 3.09 (*ddd*, *J* = 4.0; 5.0; 6.8 Hz, 1H, H-7), 3.14 (*dd*, *J* = 4.0; 8.2 Hz, 1H, H-6), 4.23 (*dd*, *J* = 7.5; 8.2 Hz, 1H, H-5), 6.06 (*dt*, *J* = 1.9; 9.7 Hz, 1H, H-2), 6.94 (*dt*, *J* = 4.3; 9.7 Hz, 1H, H-3); NMR ^13^C (CDCl_3_, 125 MHz) Δ_C_ 13.8 (C-12); 22.3 (C-11); 25.9 (C-9); 27.3 (C-7); 27.6 (C-8); 31.4 (C-10); 56.2 (C-6); 57.5 (C-7); 74.5 (C-5); 121.4 (C-2); 144.5 (C-3); 162.6 (C-1).

Epoxide (**3a**) (yield: 28%). IR (KBr) ν^max^/cm^−1^: 2936, 2847, 1734 (C = O), 1379, 1239 (C = C), 1062 (C-O-C),808 cm^−1^. ^1^H NMR (CDCl_3_, 500 MHz): Δ_H_ 0.90 (*t*, 3H, H-12), 1.35–1.60 (*m*, 8H, H-8, H-9, H-10, H-11), 2.35 (*dddd*, *J* = 1.1; 4.2; 5.9; 18.3 Hz, 1H, H-4a), 2.53 (*ddt*, *J* = 2.6; 11.7; 18.3 Hz, 1H, H-4b), 3.03 (*ddd*, *J* = 4.1; 4.3; 7.6 Hz, 1H, H-7), 3.18 (*dd*, 4.3; 7.8 Hz, 1H, H-6), 4.32 (*ddd*, *J* = 4.2; 7.8; 11.7 Hz, 1H, H-5), 6.06 (*ddd*, *J* = 1.1; 2.6; 9.7 Hz, 1H, H-2), 6.89 (*ddd*, *J* = 2.6; 5.9; 9.7 Hz, 1H, H-3); NMR ^13^C: (CDCl_3_, 125 MHz) Δ_C_ 13.8 (C-12); 22.4 (C-11); 25.9 (C-4); 26.5 (C-9); 28.1(C-8); 31.4 (C-10) 55.7(C-7); 56.8 (C-6); 77.4 (C-5); 121.6 (C-2); 143.6 (C-3); 162.8 (C-1).

### Synthesis of Compound Diol Argentilactone (2*H*-Pyran-2-one, 6-(1, 2-dihydroxyheptyl)-5,6-dihydro-, [6*R*-(1*S*, 2*R*)]) (4)

Epoxy argentilactone (0.072 g; 0.34 mmol) was treated with 2 mL of HClO_4_ aqueous solution for 12 h at room temperature. The reaction was neutralized with NaHCO_3_ and extracted with EtOAc, dried, and evaporated, yielding 0.052 mg (66.5%). IR (KBr): 3361, 2924, 2854, 1708, 1385, 1259, 1075, 822 cm-1. Diol argentilactone: [α]_D_: −82° (*c* 1.2, CHCl_3_). ^1^H NMR (CDCl_3_, 500 MHz), Δ_H_: 0.90 (*t*, 1H, H-12), 1.31–1.61 (*m*, 8H, H-8, H-9, H-10 and H-11), 2.57 (*m*, 2H, H-4), 3.63 (*dd*, *J* = 2.6, 6.5 Hz, 1H, H-6), 3.89 (*ddd*, *J* = 2.6, 4.4, 8.7 Hz, 1H, H-2), 4.49 (*m*, 1H, H-5), 6.09 (*dt*, *J* = 2.0, 9.7 Hz, 1H, H-2), 6.96 (*ddd*, *J* = 4.4, 3.5, 9.7 Hz, 1H, H-3); ^13^C NMR (CDCl_3_, 125 MHz), Δc: 13.9 (C-12), 22.6 (C-11), 25.3 (C-10), 25.5 (C-4), 31.7 (C-9), 33.6 (C-8), 69.2 (C-7),74.1 (C-6), 77.9 (C-5),120.9 (C-2), 146.1 (C-3), 164.1 (C-1).

### *P. Lutzii Pb*01 and Culture Conditions

*P. lutzii Pb*01 (ATCC-MYA-826) was previously investigated in our laboratory [14], [15]. The fungus was cultivated on agar medium (1.0% w/v peptone, 0.5% w/v yeast extract, 0.3% w/v proteose peptone, 0.5% w/v beef extract, 0.5% w/v NaCl, 4% w/v glucose, and 1.4% w/v agar, pH 7.2) [53] at 36°C or 23°C for the growth of the yeast phase or mycelium, respectively.

### Culture and Cell Viability

*P. lutzii Pb*01 yeast cells were sub-cultured every 7 days in solid Fava-Netto’s medium at 36°C. For viability experiments, yeast cells were kept in liquid McVeigh Morton (MMcM) chemically defined medium [54] for 6 h at 36°C and viability of the cells determined by counting viable cells in a Neubauer chamber. All experiments were performed in triplicate.

### Transition from Mycelium to Yeast

The differentiation from mycelium to yeast was performed in Mc Veigh and Morton (MMcM) liquid minimal medium [54] containing glucose (0.11 M) or acetate (0.28 M) as the carbon source. The cultivation temperature was changed from 23°C to 36°C to allow the mycelium to yeast transition. The cells were previously grown in liquid medium for 18 h before changing the incubation temperature, which was maintained for 10 days. The appearance of yeast cells was monitored using a Neubauer chamber.

### Sensitivity and Inhibition Assay

Inhibition assays were performed by broth macro dilution method according to NCCLS M27-A2 guidelines [55] with modifications. For the inhibition assays, yeast cells in their exponential growth phase were kept on solid MMcM for seven days at 36°C and inoculated in liquid MMcM medium. Sterile stock solution of the argentilactone, reduced argentilactone, epoxy argentilactone and diol argentilactone were freshly prepared in water and dimethyl sulfoxide (DMSO). Serial dilutions from stock solutions were prepared with sterile MMcM medium as the diluent, to yield final compound concentrations ranging from 9 to 72 µg/mL. Drug-free controls were included. Inocula concentrations were determined spectrophotometrically at 520 nm. The mixture was vortexed to disperse the aggregated cells. *P. lutzii Pb*01 was grown at 36°C, 200 rpm shaking for 10 days. For the sensitivity assay, 7-day-old *P. lutzii Pb*01 yeast cells were grown in liquid MMcM overnight at 36°C. Samples containing 10^5^, 10^4^, and 10^3^ cells were spotted onto MMcM agar medium supplemented with argentilactone, reduced argentilactone, epoxy argentilactone, or diol argentilactone at concentrations of 9 µg/mL, 18 µg/mL, 36 µg/mL, or 72 µg/mL. Control plates were did not include inhibitor. The plates were incubated for 6 days at 36°C before being photographed.

### Protein Extraction from *P. Lutzii Pb*01

*P. lutzii Pb*01 yeast cells grown for 24 h in liquid minimal MMcM medium containing glucose or acetate as the carbon source were centrifuged for 5 min at 2.500×*g*. The proteins were extracted as previously described [14]. The cells were washed with sterilized water and frozen in liquid nitrogen. The material was ground to a fine powder; resuspended in 500 µL of 50 mM potassium phosphate buffer (pH 7.0) supplemented with 1 mM dithiothreitol (DTT) and vigorously mixed with glass beads for 20 min at 4°C. The cell debris was removed by centrifugation for 15 min at 4°C and 5,000×*g*, and the supernatant was centrifuged for 15 min at 4°C and 12,000×*g*. The cell-free extract was used for the ICL activity assays or stored at −20°C for further analyses. Quantification of the protein content was performed according to Bradford [56].

### Heterologous Expression and Purification of Recombinant *Pb*ICL Protein

Recombinant *Pb*ICL protein was obtained as described by Cruz [14]. Briefly, *Pbicl* cDNA was inserted into the pET-32a (+) expression vector (Novagen, Inc,). The resulting plasmid was transformed into *E. coli* BL21 C43 (DE3) cells, and expression was induced at an A_600_ of 0.6 by the addition of 1 mm (final concentration) isopropyl thio-β-D-galactoside (IPTG) (Sigma-Aldrich). After induction, the cells were incubated for another 2 h at 36°C with shaking at 200 rpm. The cells were harvested by centrifugation at 10,000×*g* for 5 min at 4°C and resuspended in 1 × NaCl/Pi buffer. After incubation for 30 min with 100 µg/mL lysozyme, the cells were lysed by extensive sonication. The sample was centrifuged at 4°C and 8,000×*g* for 15 min, and the supernatant, which contained the soluble protein fraction, was collected. His-tagged ICL was purified using the Ni-NTA Spin Kit (Qiagen), and the tags were subsequently removed by the addition of EKMax™ enterokinase (Invitrogen).

### Determination of Enzymatic Activity

ICL activity was determined according to Cruz *et al*. [14]. Reactions were performed in 1 mL assay volume containing 2 mM threo-D,L-isocitrate (effective concentration of threo-D-isocitrate 1 mM), 2 mM MgCl_2_, 10 mM phenylhydrazine HCl, 2 mM dithiothreitol, and 50 mM Tris HCl buffer (pH 7.0). The product glyoxylate-phenylhydrazone was followed at 324 nm using an extinction coefficient of 16.8 mM^−1^.cm^−1^. One unit of enzyme activity was defined as the formation of 1 µmol of glyoxylate-phenylhydrazone per minute using threo-D,L-isocitrate as the substrate. The specific activities were given as U.mg^−1^ protein.

The compound concentrations were 10 µM, 20 µM, 30 µM, and 40 µM for recombinant *Pb*ICL and 30 µM, 40 µM, 50 µM, 60 µM, 70 µM, 80 µM and 90 µM for native *Pb*ICL. 3-nitropropinate (50.7 µM) was used as positive control [16]. To the assays of specific activity of *Pb*ICL from mycelium, transition, and yeast, the cells were cultured for 10 days on 23°C and 36°C, and were used 18 µg/mL of argentilactone.

### Receptor Preparations

The 3D structure of *Pb*ICL (537 aa) has not yet been resolved experimentally. Therefore, the amino acid sequences were compared against the PDB using the BlastP program [46] to obtain the highest identity. The tertiary structure was initially predicted by homology modeling using the algorithm on server ModWeb [57], with only the sequences being inserted to provide preliminary models. ModWeb uses high-identity templates of the structures deposited in PDB to determine 3D models, which are classified according to the lowest discrete optimized protein energy score. The quality of the structures predicted at this stage was measured using the NIH-MBI laboratory server [58] with ERRAT [47], which provides an overall quality factor based on the statistics of the non-bonded interactions between different atom types. This factor is expressed as the percentage of the protein for which the calculated error value falls below the 95% threshold. A Ramachandran plot for each protein was generated on the RAMPAGE web server [59], and Verify 3D was used to evaluate the amino acid environment [60]. Percentages of the helical and sheet contents were estimated using the 2Struc DSSP server [61], and Helix System [62] was used to generate the linear representation of the secondary structures.

Molecular dynamic simulations of these structures were performed to improve the relaxation and orientation of the side chains. This procedure is usually crucial in molecular docking to reproduce the structural stability of the receptor in its native environment [63]. The software GROMACS 4.1.5 [64], [65] was used to solvate the models in a cubic box with the chosen force field, and the solvent was treated explicitly (SPC water model).

The particle mesh Ewald method [66] was used to improve the treatment approaches involving the electrostatic interactions with periodic boundary conditions considered in all directions from the box. Initially, the system was neutralized by adding counter ions and then immediately subjected to minimization using the steepest descent energy. The simulations were completed when the tolerance of 1000 kJ/mol was no longer exceeded. Conformations involving high energies and overlap between the atoms were eliminated at this stage.

The first step in the equilibration of the system was energy relaxation of the solvent for 100 ps; the system was subjected to molecular dynamic only after this step. The simulations were performed for 20 ns for *Pb*ICL with a constant temperature of 300 K, 1 atm pressure, time-step of 2 fs, and without any restriction of protein conformations/segments. All information concerning the trajectory of these times were collected every 5 ps. The equilibration of the trajectory was evaluated by monitoring the equilibration of quantities, such as the RMSD of the non-hydrogen atoms, with respect to the initial structure. The analysis of the total energy, potential energy, and kinetic energy were all obtained using GROMACS analysis tools. The RMSD values between the final and template structures also helped to identify the common segments corresponding to the structurally conserved region.

The program g_cluster (GROMACS tool) was used to determine the conformations that best represent the structures of the entire trajectory obtained during the simulation. The algorithm *gromos*, as described in Daura [67], was selected for this purpose. A cutoff = 0.3 nm for the clusters was used considering the profile of the RMSD observed in Figure 4 (evolution of RMSD). The clusters were determined using the non-hydrogen atom RMSD values. The average structure of the entire trajectory was also determined using the g_rms algorithm [68]. The first 10 ns of the trajectory were not used to determine the average structures of *Pb*ICL. Aiming to prepare the structures for docking, all of the water molecules were removed from the selected structures. ASA was determined using the function of get_area pymol [69], which calculates the surface area in square Angstroms of the selection. The option dot_density = 3 was selected to improve the accuracy of the ASA estimates.

### Ligand Preparations

The 3D structures of the compounds were generated using GlycoBioChem PRODRG2SERVER [70]. After conversion to PDB format, the charges and non-polar hydrogen atoms were added using the prepare_receptor4.py script from MGLTools [71], and pdbqt files (format for Autodock) containing the individual compounds and receptor were created. The torsions of the compounds were also considered and included in the pdbqt files using MGLTools. The conformations of those files were used as the initial conformations for the AutoDock Vina [51] program for the molecular docking simulations [51].

### Molecular Docking

AutoDock Vina [51] is a method that uses derivatives of the scoring function with respect to its arguments to sample conformations of the protein surface. This method allows the rapid identification of the minimum of the function within the defined grid (also includes a local search stochastically). However, to increase the efficiency of the sampling, 1000 independent simulations were performed in addition to those already performed when AutoDock Vina [51] uses *exaustiveness* = 8.

AutoDock Vina [51] uses a conformation-independent function, *g*, given by
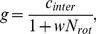
(1)where *N*rot is the number of active rotatable bonds between heavy atoms in the ligand and *w* is the associated weight. Because of the exhaustive sampling performed for each ligand, in this work, we consider the Fscore given by
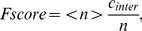
(2)where n is the number of ligand atoms (except hydrogens) and <n> is the average number of atoms involved (except hydrogens) among all the compounds under comparison. The values of g estimated from AutoDock Vina [51] (Eq. 1) were recalculated by Eq. 2, and an Fscore histogram was built for each ligand.

### Intermolecular Interactions in the Protein-ligand Complexes

The conformations of the protein-ligand complexes with the lowest Fscore values extracted from AutoDock Vina [51] were selected for a new molecular dynamic to determine the principal intermolecular iterations when the ligand and receptor are solvated in the same complex. The topology of the ligands was generated using GlycoBioChem PRODRG2SERVER [70], and the topology of the receptor was generated using the GROMOS96 force field.

The minimization of the complex followed the same protocol used for the receptor in section 4.11 (receptor preparation). A time of 100 ps was required for the equilibration; the system was subjected to molecular dynamic only after this step. The simulations were performed for 2 ns for *Pb*ICL at a constant temperature of 300 K, 1 atm. pressure, time-step of 2 fs, and considering restriction of the protein conformations.

### Ligand Solvation Free Energy

In this work, the ligand solvation free energy was determined when the ligand was taken from state A (solvated) to another state, B (*in vacuum*). The free energy differences were determined directly by the sample properly states involved in the transition between A and B by mapping states that are close enough to connect the states [72]. By using free energy perturbation methods, it is possible to couple the interaction strength between ligand and solvent to a variable, λ,
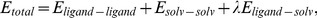
(3)and slowly turn λ from 1 to 0. This is equivalent to slowly turning off or placing the ligand in a vacuum, i.e., slowly turning off the interactions between the solvent and ligand. The number of λ points used to describe the transformation from state A (λ = 0) to state B (λ = 1) and the free energy difference ΔG_AB_ are functions of the coupling parameter λ, which can be determined according to the fluctuations occurring in Eq. 3, i.e., determining ∂E/∂λ over the entire range between adjacent values of λ. Simulations were performed at different values of λ to obtain a ∂E/∂λ curve from which ΔG_AB_ was derived for each ligand.

The analyses in this study were performed using the tool g_BAR GROMACS version 4.5, which employs the Bennett acceptance ratio (BAR) method for calculating free energy differences [72]. We considered dλ = 0.05 sufficient to achieve a very good accuracy, thus resulting in a total of 30 simulations for each ligand (λ = 0, λ = 0.05, λ = 0.1, λ = 0.15, λ = 0.20). The protocol for each molecular dynamic simulation involving the estimated ligand free energy followed the scheme of J. Lemkul [73].

### Statistical Analysis

The *Pb*ICL activity experiments were performed in triplicate, and the results are presented as the means (±) standard deviation, Tukey's test was applied to compare the values from enzymatic assays with treated and untreated the compounds and viability assay. A one-way ANOVA multiple test was applied to compare the values obtained from minimal inhibitory concentration and transition. Statistical comparisons were performed using STATISTICA software version 8.0 [74] Significance was accepted at *p*<0.05.

## Supporting Information

Figure S1 **Fscore of argentilactone obtained in the binding pocket of mutated** ***Pb*** **ICL.** Vertical bars represent the total number of hits observed for each Fscore (total of 1000 independent simulations). Fscore values refer to modes of lower energies observed in the simulations with AutoDock Vina involving mutated and non-mutated binding pocket. Green bars correspond to non-mutated *Pb*ICL (native *Pb*ICL) Fscore. Bars in blue, red and cyan correspond to mutations involving ASN53, GLY235 and LYS47 residues.
(TIFF)
