# Supplementary figures and images for: Inhibition of Paracoccidioides lutzii Pb01 Isocitrate Lyase by the Natural Compound Argentilactone and Its Semi-Synthetic Derivatives
Source: PLoS One. 2014 Apr 21;9(4):e94832. doi: 10.1371/journal.pone.0094832 (PMC3994062; doi:10.1371/journal.pone.0094832)

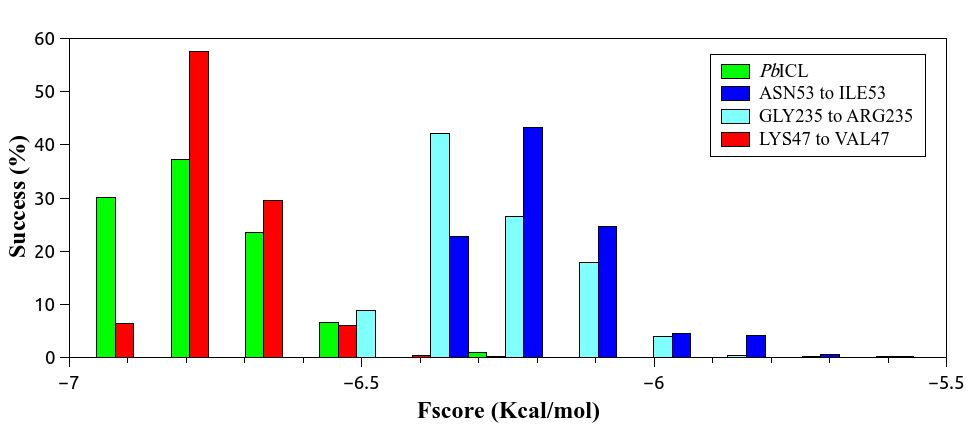

Supplement: Figure S1 — Fscore of argentilactone obtained in the binding pocket of mutated Pb ICL. Vertical bars represent the total number of hits observed for each Fscore (total of 1000 independent simulations). Fscore values refer to modes of lower energies observed in the simulations with AutoDock Vina involving mutated and non-mutated binding pocket. Green bars correspond to non-mutated PbICL (native PbICL) Fscore. Bars in blue, red and cyan correspond to mutations involving ASN53, GLY235 and LYS47 residues. (TIFF) [file pone.0094832.s001.tiff]
